# Tracheomediastinal Fistula in a Patient With Lung Adenocarcinoma and Its Treatment With Argon Plasma Coagulation: A Case Report

**DOI:** 10.1097/MD.0000000000000156

**Published:** 2014-11-14

**Authors:** Mehtap Ucer, Cetin Ordu, Kezban Nur Pilancı, Levent Dalar

**Affiliations:** From the Department of Internal Medicine (MU); Department of Medical Oncology (CO, KNP); and Department of Pulmonary Medicine (LD), School of Medicine, Istanbul Bilim University, İstanbul, Turkey.

## Abstract

Tracheomediastinal fistula is a rare complication that occurs during the course of lung cancer. The fistula connects the airways to the mediastinum and is often associated with lymphoma. Clinical data on tracheomediastinal fistulas are limited to case reports. Tracheal stenting, pericardial and omental patch closure, and muscle flap closure can be performed to repair such fistulas. We herein report a case of tracheomediastinal fistula in a 47-year-old man.

The main symptoms were shortness of breath and a feeling of fullness in the neck. Thoracic magnetic resonance imaging revealed an approximately 57 × 16 × 20 mm multiloculated cystic lesion with air density located in the upper mediastinum of the right paratracheal region and a fine fistula tract at this level. The main diagnosis was primary lung adenocarcinoma-related mediastinal lymphadenomegaly with a tracheomediastinal fistula.

The patient underwent fistula opening on the trachea, which was then coagulated and sealed using argon plasma coagulation.

The patient is currently asymptomatic and doing well 8 months after the intervention.

## INTRODUCTION

Tracheomediastinal fistula is a rare complication that occurs during the course of lung cancer. These airway fistulas are often associated with the esophagus or pleural space. Mediastinal fistulas are extremely rare. The fistulas are often associated with lymphoma. Clinical data on tracheomediastinal fistula are limited to case reports. Tracheal stenting, pericardial and omental patch closure, autologous stem cell therapy, and muscle flap closure can be performed to repair these fistulas.^1–6^

We herein describe, to our knowledge, the first case of a tracheomediastinal fistula treated using argon plasma coagulation (APC).

## CASE REPORT

A 47-year-old man with unknown previous conditions underwent cranial magnetic resonance imaging (MRI) after having experienced tonic–clonic seizures in November 2012. MRI revealed nodular lesions and vasogenic edema. Positron emission tomography-computed tomography revealed a 44 × 30 mm primary lesion located in the right hilar region as well as hypermetabolic lymphadenomegaly of the lymph nodes in the mediastinum of the right paratracheal region, aorticopulmonary window, and subcarinal and right hilar regions. The biopsy specimen obtained during bronchoscopy revealed adenocarcinoma. Radiotherapy was planned because of the presence of more than 20 cranial metastases. Palliative 3-dimensional conformal radiotherapy was delivered for 15 days (total of 30 Gy in 2-Gy fractions).

The cranial lesions regressed following whole-brain radiotherapy. The patient then underwent 6 cycles of docetaxel at 120 mg + cisplatin at 120 mg, followed by 8 cycles of pemetrexed and 3 cycles of cisplatin and gemcitabine.

**FIGURE 1:**
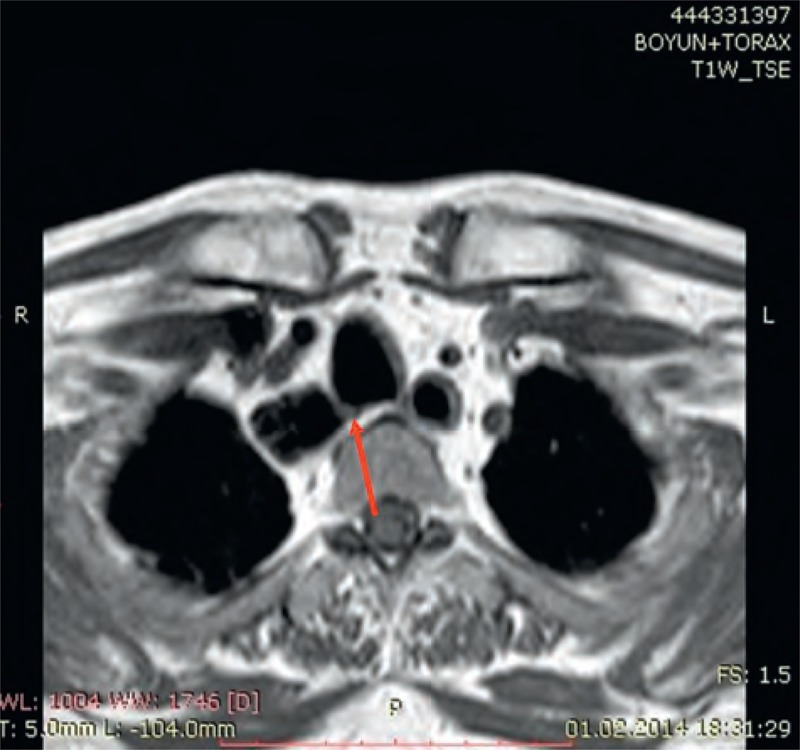
Thoracic magnetic resonance imaging revealed mediastinal cystic lesions communicating with the trachea (arrowhead indicates the fistula tract).

One year following initial therapy and 3 months after the last dose of chemotherapy, the patient presented with shortness of breath and a feeling of fullness in the neck. Thoracic MRI revealed an approximately 57 × 16 × 20 mm multiloculated cystic lesion with air density located in the upper mediastinum of the right paratracheal region and a fine fistula tract at this level associated with the trachea from its right posterolateral aspect (Figure 1).

During bronchoscopy, a 1-mm fistula opening was observed in the posterior wall of the right lateral aspect, approximately 6 cm distal to the cricoid cartilage. This area was coagulated using APC. Closure of the fistula opening occurred by secondary cicatricial healing.

**FIGURE 2:**
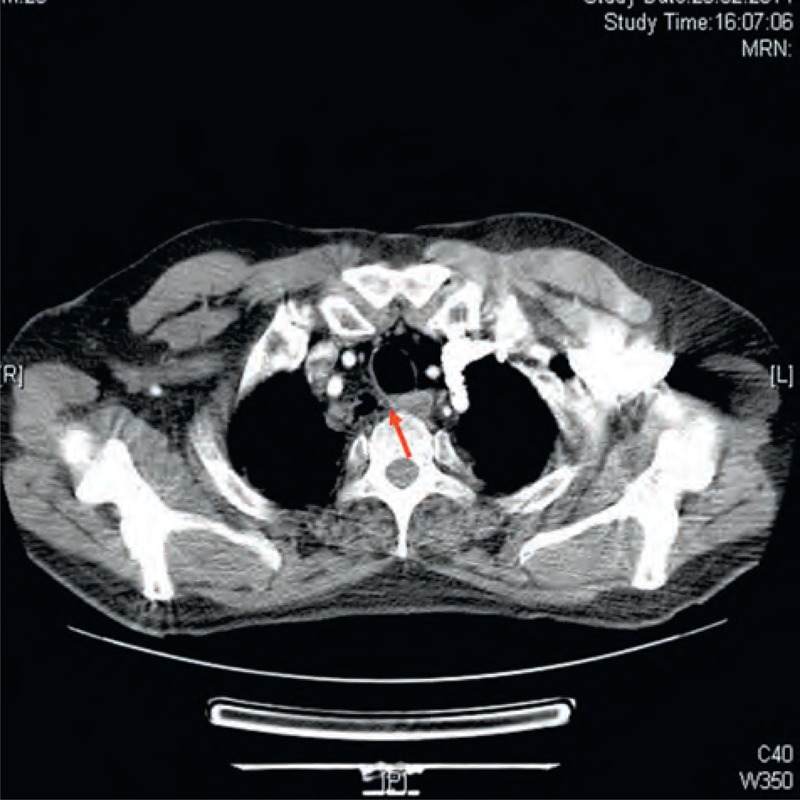
Thoracic computed tomography revealed regression of the lesion after closure of the temporalis muscle flap using argon plasma coagulation (arrow indicates that the connection between the cystic lesion and the trachea disappeared).

**FIGURE 3:**
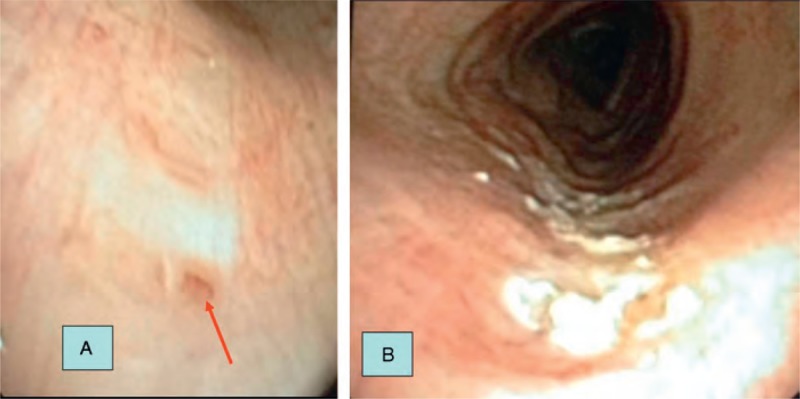
(A) Bronchoscopic appearance of the approximately 1-mm fistula opening 6 cm distal to the cricoid cartilage (arrow). (B) Control bronchoscopic appearance after argon plasma coagulation.

Control thoracic CT performed 1 week after the procedure revealed substantial improvement in the mediastinal cystic lesion (Figure 2), and control bronchoscopy confirmed closure of the fistula opening (Figure 3A and B).

The patient did not provide any consent to publish his medical records because of his identity to remain anonymous. The Ethic Committee of the School of Medicine, Istanbul Bilim University, İstanbul, Turkey, has waived the approval because this is a case report.

## DISCUSSION

Despite their rare occurrence, fistulas communicating with the bronchial tree are important causes of increased mortality and morbidity. Such fistulas most commonly communicate with the esophagus; however, they may also communicate with the pleura and mediastinum.

The underlying etiology of bronchopleural fistulas (BPFs) is variable. However, pulmonary resection, various infections, chemotherapy, and radiotherapy, in particular, are used for the treatment of lung cancer; spontaneous persistent pneumothorax and tuberculosis are commonly implicated factors.^7^

Insertion of a thoracic tube is one treatment method for BPF^7^ and is particularly preferred for BPF with high output and empyema drainage; however, such tubes can cause a loss of tidal volume, abnormal gas exchange and ventilator cycles, and a predisposition to infections.^7^

Surgical treatment of BPF involves many techniques with high success rates and low mortality, including chronic open drainage, closure with intercostal muscles, omental flap, and transsternal bronchial closure.^7^

Bronchoscopy can be employed for both diagnostic and therapeutic purposes in patients with BPF.^7^ Many occlusive agents (ethanol, silver citrate, fibrin and tissue adhesives, antibiotics, gel foams, and autologous blood patches) have been attempted to close fistula tracts during bronchoscopy. These procedures are often preferred in patients with a poor general condition who cannot tolerate surgery.^7^

Fistulas that develop between the airway and esophagus are another common form of fistulas that communicate with the airways, and these fistulas often occur in patients with inoperable tumors, for whom increasing the quality of life has become the treatment focus.^8^

The data on tracheomediastinal fistulas are mostly limited to case reports. This type of fistula often develops in association with lymphoma. Tracheal stenting, pericardial and omental patch closure, autologous stem cell therapy, and muscle flap closure can be performed to repair such fistulas.^1,2,4–6,9–15^

Huang et al^1^ used a self-expandable metallic stent for the treatment of a tracheomediastinal fistula in a patient with non-Hodgkin lymphoma. During a 7-year follow-up, they observed the healing of the fistula through tumor-specific therapy without development of mediastinitis. Shin et al^6^ used 2.0 and 0.5-cm temporalis muscle flaps (TMFs) in a patient with mid-thoracic esophageal cancer treated with a self-expanding tracheal stent. This provided rapid symptomatic recovery and relief of mediastinal necrosis and pneumonia. In another report,^5^ surgical intervention was required in a patient with a 5-mm TMF because of an anterior mediastinal infection and the need for debridement; the TMF was closed with a muscle flap with multiple pedicles. Diaz-Agero et al^2^ used autologous fat tissue stem cells because of the risk of open surgery and bleeding in a patient monitored for non-small cell lung cancer with a 10-mm TMF and reported a successful outcome over a 2-year follow-up period.

Most fistulas that occur in association with lung cancer are BPF that develop as a complication of lung resection. Most tracheomediastinal fistulas develop in association with shrinking lymph nodes after radiotherapy or chemotherapy, particularly in patients with Hodgkin lymphoma. In this case, the tracheomediastinal fistula that developed during follow-up for adenocarcinoma was considered to be related to a lymph node located in the right paratracheal region; it was detected at the time of the initial diagnosis and showed necrosis and consolidation after chemotherapy. The necrosis of the lymph node that was previously attached to the tracheal wall was regarded as the cause of fistula formation.

Because the current patient was found to have a fistula with a 1-mm diameter on bronchoscopy and his general condition was poor, the fistula tract was closed using an endoscopic technique. The fistula opening was circumferentially coagulated using 40-W APC (ERBE, Tübingen, Germany), and closure of the opening was observed 1 week after the procedure.

Closure of a fistula tract using APC was previously described in a patient with a BPF, and the selection of this method was similarly based on the small diameter of the fistula.^3^

In conclusion, to our knowledge, this is the first successful use of APC for closure of a tracheomediastinal fistula in a patient with adenocarcinoma.
